# Seipin overexpression attenuates cerebral ischemia‐reperfusion injury via preventing apoptosis and autophagy

**DOI:** 10.1002/brb3.3195

**Published:** 2023-10-27

**Authors:** Xiaoxi Zhu, Xiaoqiong An, Ming Chen, Dongfen Guo, Peng Xie, Bi Wang, Zhi Huang, Wenfeng Yu

**Affiliations:** ^1^ Key Laboratory of Molecular Biology School of Basic Medical Science of Guizhou Medical University Guiyang City China; ^2^ Cell engineering Laboratory Affiliated Hospital of Zunyi Medical University Zunyi City China

**Keywords:** apoptosis, autophagy, CI/RI, seipin

## Abstract

**Background:**

Ischemic cerebrovascular disease (ICVD) is one of three fatal diseases in humans, along with heart disease and malignant tumors. Cerebral ischemia/reperfusion injury (CI/RI) is the primary cause of ICVD. Recently, seipin was reported to be crucial for lipid droplet formation, hepatic steatosis, and axonal atrophy. However, the function and mechanism of seipin in CI/RI has not been explicitly stated.

**Methods:**

Oxygen–glucose deprivation/reoxygenation (OGD/R) hippocampal neuron cell line (HT‐22) and middle cerebral artery occlusion (MCAO) in rats were established. The levels of apoptosis‐ and autophagy‐related proteins and seipin were confirmed by western blot. Cell proliferation was assessed with CCK‐8, and ischemic infarction and pathological structure were monitored by TTC and H&E staining, and tissue apoptosis was assessed through TUNEL assay.

**Results:**

The proliferative activity was decreased, and apoptosis and autophagy pathways could also be induced in the OGD/R HT‐22 cells. Seipin overexpression accelerated viability and inhibited apoptosis and autophagy in the OGD/R HT‐22 cells. Moreover, the data revealed that seipin overexpression could also attenuate cerebral infarction, apoptosis. Autophagy pathways could be repressed by seipin in the MCAO rats.

**Conclusion:**

Seipin has a protective role against CI/RI in rats, and its mechanism might be relevant to the suppression of apoptosis and autophagy, suggesting that seipin might be a latent target for CI/RI.

## Graphical abstract

Seipin induces proliferation of OGD/R HT‐22 cells Seipin prevents apoptosis and autophagy of OGD/R HT‐22 cells Seipin attenuated cerebral infarction in MCAO model rats Seipin inhibits apoptosis and autophagy in MCAO model rats.

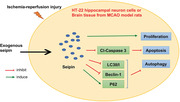

## 1 INTRODUCTION

Cerebrovascular disease (CVD) is a hackneyed chronic disease in senior citizens, and the incidence of ischemic CVD is the highest, with approximately 80% (Caprio & Sorond, 2019). Cerebrovascular disease is mainly caused by reduced cerebral blood flow due to vascular embolism and insufficient blood oxygen supply to the brain tissue (Goldstein, 2020). Therefore, it is important to restore blood reperfusion in ischemic areas as soon as possible by thrombus dissolution or mechanical recanalization (Liao et al., 2020; Mazur et al., 2021). However, under certain circumstances, the return of blood flow after ischemia can cause dysfunction and tissue damage, known as CI/RI (Wu et al., 2018). The mechanism of CI/RI is complex, involving oxygen‐free radical accumulation, mitochondrial damage, inflammatory response, calcium overload, cell apoptosis, and excessive release of excitatory amino acids, among others (Liao et al., 2020; Liu et al., 2021). Currently, the drugs adopted to treat CI/RI often have large toxic side effects, single mechanisms, and poor effect (Guan et al., 2021; Hausburg et al., 2020). Therefore, it is important to investigate the potential functions and mechanisms of genes related to CI/RI therapy.

Seipin is a transmembrane protein in the endoplasmic reticulum (ER) and contains two transmembrane domains (Craveiro Sarmento et al., 2018). Previous studies have shown that seipin plays a protective role in lipid homeostasis and in the maintenance of lipid droplet morphology (Jin et al., 2020; Rao & Goodman, 2021). A lack of seipin protein can impair adipocyte homeostasis and cause lipid dysmetabolism (Jackson, 2019). Loss of seipin function in the testis has also been shown to be associated with teratozoospermia (Jiang et al., 2014). As research progressed, a study also reported that seipin was upregulated in the brain (Ebihara et al., 2015). Abnormal seipin can cause nerve involvement, suggesting that seipin plays a key role in brain neurons (Liu et al., 2016). Seipin is a key regulatory molecule in neuronal disease (Wang et al., 2018a). Normally, seipin is upregulated in certain areas of the brain (the spinal cord, frontal cortex, and areas involved in regulating energy balance) (Liu et al., 2016). Seipin missense mutations have also been proven to cause dominant inherited motor neuron disease (seipin seipinopathy) (Chang et al., 2019). Therefore, seipin may make a significant contribution to neurological diseases. However, the effect and mechanism of seipin on CI/RI function remain unclear.

Here, we first established OGD/R hippocampal neuron cell line (HT‐22) model and MCAO rats and further examined the expression change of seipin. Functionally, we further verified the influence of seipin overexpression on proliferation, apoptosis, and autophagy in the OGD/R HT‐22 model cells and MCAO rats as well as the effects of seipin on cerebral infarction and the pathogenic structure of MCAO rats. Therefore, we propose that seipin may be a potential target for CI/RI therapy.

## 2 MATERIAL AND METHODS

### 2.1 Animals

Healthy Sprague–Dawley (SD) rats of SPF grade (male, 12−14 weeks, 240−260 g) were obtained from the Guizhou Laboratory Animal Engineering Technology Center. This experiment was approved by Experimental Animal Care and Usage Committee of Guizhou Medical University (approval number 2100344). All animal procedures were conducted with the NIH guidelines. The rats were kept in the laboratory at 22−24°C, with alternating light/dark light conditions for 12 h, clean water, and plenty of food. All rats were fed adaptively for one week before surgery (Xia et al., 2021).

### 2.2 Establishment of MCAO model

Before modeling, the rats were fasted for 12 h, followed by MCAO. In reference to the previous research (Zhou et al., 2018), rats were anesthetized by applying 1% pentobarbital sodium (50 mg/kg), and the right common carotid artery (CCA) was isolated. We then conducted ligation with a 5‐0 stylolite from the external carotid artery (ECA) to the internal carotid artery (ICA). After 2 h, the ligation site was opened for 12 h to restore cerebral blood circulation, and the incision was immediately sutured. The rats underwent sham surgery (the rats received the same procedure but did not ligate the arteries). Rats with massive bleeding, subarachnoid hemorrhage, and premature death were excluded after cerebral ischemia‐reperfusion injury. Finally, 40 (*n* = 10 per group) male Sprague–Dawley rats were used in the experiment.

### 2.3 Seipin overexpression in rats

After anesthesia, the head of the rats was first attached to a stereoscope, and the scalp was cut open to expose the skull. Then, a bone drill needle was applied to make a small hole in the lateral ventricle (from the mid‐line: 0.5 mm left; from the bregma: 0.8 mm anterior; from the surface: 2.5 mm ventral). Then, the lateral ventricle of the rats was injected with lentivirus‐packed seipin (10 μL, 1 × 10^9^ TU/mL, Ruibio, China) at a rate of 1 μL/min for 10 min, and the needle was left for 2 min. After the scalp was sutured, the rats were routinely fed for three weeks, and subsequent experiments were conducted (Zhou et al., 2018).

### 2.4 Neurologic score

The neurological function score was conducted with reference to the Siegal method, which is divided into 5 levels (Siegal et al., 1988). Level 0 was normal; level 1 had weakness in tail‐flicking; level 2 had weakness in the hind limbs with mild difficulty in walking; level 3 had weakness in the hind limbs with significant instability in walking; level 4 had unstable standing but was able to move the hind limbs; level 5 had paralysis with no voluntary movement of the hind limbs.

### 2.5 TTC staining

After decapitation, the lower brain stem, olfactory bulb, and cerebellum of the rats were removed, and the brain was frozen, and cut into coronal slices. The sections were exposed to 1% TTC (Servicebio, G1017) for 30 min and fixed with 10% neutral formaldehyde for 2 h. Subsequently, the sections were photographed, and the infarcted areas were tested by applying Image‐Pro Plus (Zhang et al., 2021).

### 2.6 Construction of the OGD/R cell model

HT‐22 cell line were from BeiNa Culture Collection (Beijing, China) and incubated in DMEM (Gibco, 11965092) with 10% FBS (Sigma, 10099141C) and 2 mM glutamine at 37°C, 5% CO_2_. Then, the HT‐22 cells were grown in glucose‐free DMEM (Gibco) with 1 % O_2_, 94 % N_2_, and 5 % CO_2_ at 37°C for 1, 2, 4, 8, and 12 h. Subsequently, the HT‐22 cells were placed to normal DMEM with 95% air and 5% CO_2_ for 24 h (Pan et al., 2022). The HT‐22 cells under normal oxygen conditions were applied as the control.

### 2.7 Cell transfection

To overexpress seipin, we cloned the seipin gene into pcDNA‐3.1 vectors to generate the recombinant plasmid through molecular cloning. Then, OGD/R HT‐22 cells were addressed with seipin overexpression plasmid or empty vector using Lipofectamine™ 3000 (Invitrogen, L3000001) (Zeng et al., 2022).

Transfection was performed with mCherry–GFP–LC3B (Beyotime, C3011) adenovirus in HT‐22 cells according to the manufacturer's instructions. Cells were inoculated into 24‐well plates for 24 h, and then transfected with mCherry–GFP–LC3B adenovirus for 12 h at 37°C. After OGD treatment, the GFP and mRFP puncta were viewed under a confocal microscope (Zhang et al., 2020a).

### 2.8 CCK‐8

Processed HT‐22 cells (1 × 10^4^ cells/well) were uniformly added to 96‐well plates. Then, 15 μL of CCK‐8 (Dojindo; CK04) was added at 48 h. After 3 h, the absorbance was monitored with a microplate reader (Bio‐Tek Epoch) at 450 nm (Huang et al., 2022).

### 2.9 Flow cytometry

The treated HT‐22 cells (1 × 10^6^ cells/well) were inoculated in 6‐well plates and cultured to the normal growth stage. Subsequently, the HT‐22 cells were supplemented with 30 μM EdU solution (RIBIBIO, C10338‐1) and incubated for 2 h at 37°C. After collection, the cells were suspended, immobilized, and subjected to multiple processes, including neutralization with 2 mg/mL glycine (5 min), permeation with 0.5% TritonX‐100 (10 min), and 1X Apollo® solution (10 min). Flow cytometry was conducted immediately after staining (Zhou et al., 2020).

### 2.10 H&E staining

Brain tissue was collected, fixed, dehydrated (gradient alcohol), made transparent (xylene I and xylene II), and embedded in paraffin. The paraffin tissue was then sectioned (approximately 5‐μm thick) and baked at 65°C for 3 h. After dewaxing and hydration, the slices were subjected to several treatments, including hematoxylin staining (Servicebio, G1076), 1% hydrochloric acid ethanol differentiation, ultrapure water immersion, and eosin staining. The slices were processed again with graded alcohol, xylene, and neutral gum solutions. Finally, the staining results were examined (Ghotbeddin et al., 2020).

### 2.11 TUNEL

Apoptosis‐positive cells in the rat brain group were detected using TUNEL staining kits according to the manufacturer's instructions (Servicebio, G1507). Brain sections of each group were routinely dewaxed for 10 min and dehydrated with gradient alcohol. Then the sections were addressed with proteinase K (20 mg/L) for 15 min and 0.5% H_2_O_2_ for 20 min. After PBS washing, the sections were incubated with TUNEL (50 μL) for 60 min in a wet chamber at 37°C. Then the sections were disposed of fluorescein antibody. Then the sections were subjected to multiple procedures including diaminobenzidine (DAB) incubation, hematoxylin treatment, xylene, gradient ethanol dehydration, and neutral resin sealing. An inverted microscopy was adopted to obtain tissue images (Feng et al., 2020).

### 2.12 RT‐qPCR

The processed HT‐22 cells and brain tissues (10 mg) were collected, and 500 μL TRIzol (Invitrogen, 15596) was added to isolate the total RNAs. After centrifugation at 12,000 × *g* for 10 min, the supernatant was mixed with 200 μL chloroform in a centrifuge tube and centrifuged at 12,000 × *g* for 10 min at 4°C. The supernatant was then mixed with 600 μL of isopropyl alcohol in a new 1.5 mL centrifuge tube and centrifuged at 12,000 × *g* for 10 min at 4◦C. After discarding the supernatant, the precipitate was rinsed with 1 mL of 75% absolute ethanol (750 μL absolute ethanol and 250 μL of DEPC water) and then, 1 mL of absolute ethanol. After centrifugation at 12,000 × *g* for 5 min at 4°C, the supernatant was discarded and the RNA was resuspended in 40 μL of DEPC water for storage at −80°C prior to analysis. Reverse transcription was conducted using a Reverse Transcription Kit (Takara, 639505). cDNA was generated by reverse transcription at 42°C for 60 min and 95°C for 5 min. The amplification reaction was conducted using SYBR Green PCR Master Mix (Applied Biosystems, A46012). The reaction system was prepared with 10 μL of SYBR Mixture, 1 μL of PCR Forward Primer (10 μM), 1 μL of PCR Reverse Primer (10 μM), 1 μg of cDNA template, and ddH_2_O up to 20 μL. The qRT‐PCR conditions were as follows: 95°C for 10 min denaturation, followed by 40 cycles of 95°C for 15 s, and 60°C for 30 s. The obtained data were counted using the 2^−△△Ct^ method (Feng et al., 2020). And primer sequences were presented as follow: P62 (F: 5′‐AGG ATG GGG ACT TGG TTG C‐3′; R: 5′‐TCA CAG ATC ACA TTG GGG TGC‐3′), Beclin 1 (F: 5′‐ATG GAG GGG TCT AAG GCG TC‐3′; R: 5′‐TCC TCT CCT GAG TTA GCC TCT‐3′), LC3B (F: 5′‐TTA TAG AGC GAT ACA AGG GGG AG‐3′; R: 5′‐CGC CGT CTG ATT ATC TTG ATG AG‐3′), Seipin (F: 5′‐TGG GGC AAG AGA GAC ATG C‐3′; R: 5′‐TCT TCC ACA GGG ACG ATA CCC‐3′), β‐actin (F: 5′‐CAT TGC TGA CAG GAT GCA GA‐3′; R: 5′‐CTG CTG GAA GGT GGA CAG TGA‐3′).

### 2.13 Western blot

Total protein was detached from the processed HT‐22 cells and brain tissues using RIPA buffer (Beyotime, P0013B). Cells and brain tissues were placed in Petri dishes containing 1 mL of precooled Lysis Buffer, and homogenized. The homogenate was centrifuged at 12,000 × *g* for 20 min at 4°C. The supernatant was transferred to a precooled centrifuge tube and protein denaturation was carried out by the addition of loading buffer. Samples were boiled for 15 min at room temperature. The total protein concentration of the sample was determined using a BCA protein concentration assay kit (Beyotime, P0009). After concentration quantification, proteins were then separated by SDS‐PAGE concentrated glue and transferred to a PVDF membrane (Millipore, IPVH00010). After washing (3 × for 10 min) with Tris buffered saline‐tween (TBST), the PVDF membrane blocked in 5% skimmed milk powder for 2 h. After washing (3 × for 15 min) with TBST, the membrane was incubated (with shaking) overnight at 4°C and with primary detection antibodies. The following primary antibody were employed for WB: anti‐seipin antibody (abcam, ab106793), anti‐Cleaved Caspase‐3 (abcam, ab32042), anti‐p62 (abcam, ab207305), anti‐Beclin‐1 (Cell Signaling Technology, #3738), anti‐LC3B (Cell Signaling Technology, #2775), anti‐AIF (abcam, ab32516), anti‐Endog (abcam, ab76122), and anti‐GAPDH (abcam, ab18162). The next day, membranes were shaken at room temperature for 30 min and then washed (3 × for 15 min) with TBST. Membranes were then incubated with the secondary detection antibody diluted in blocking solution and shaken for 1 to 2 h at room temperature. Subsequently, membranes were washed (3× for 10 min) with TBST and protein bands were detected using a chemiluminescent reagent (Tanon, 180–5001). Each experiment was repeated three times using the same procedure to obtain an average value (Feng et al., 2020).

### 2.14 Statistical analysis

All data were represented as mean ± SD of three replicates. SPSS (version 22.0; SPSS Inc.) was applied for statistical analysis, and GraphPad Prism 8.0 was applied for graph drawing. Comparisons between two groups were performed using Student's *t*‐test, and multiple comparisons were performed by one‐way analysis of variance (ANOVA) carrying with a post hoc Bonferroni test. And *p* < .05 denoted statistically significance.

## 3 RESULTS

### 3.1 Identification of proliferative activity in OGD/R HT‐22 cells

**FIGURE 1:**
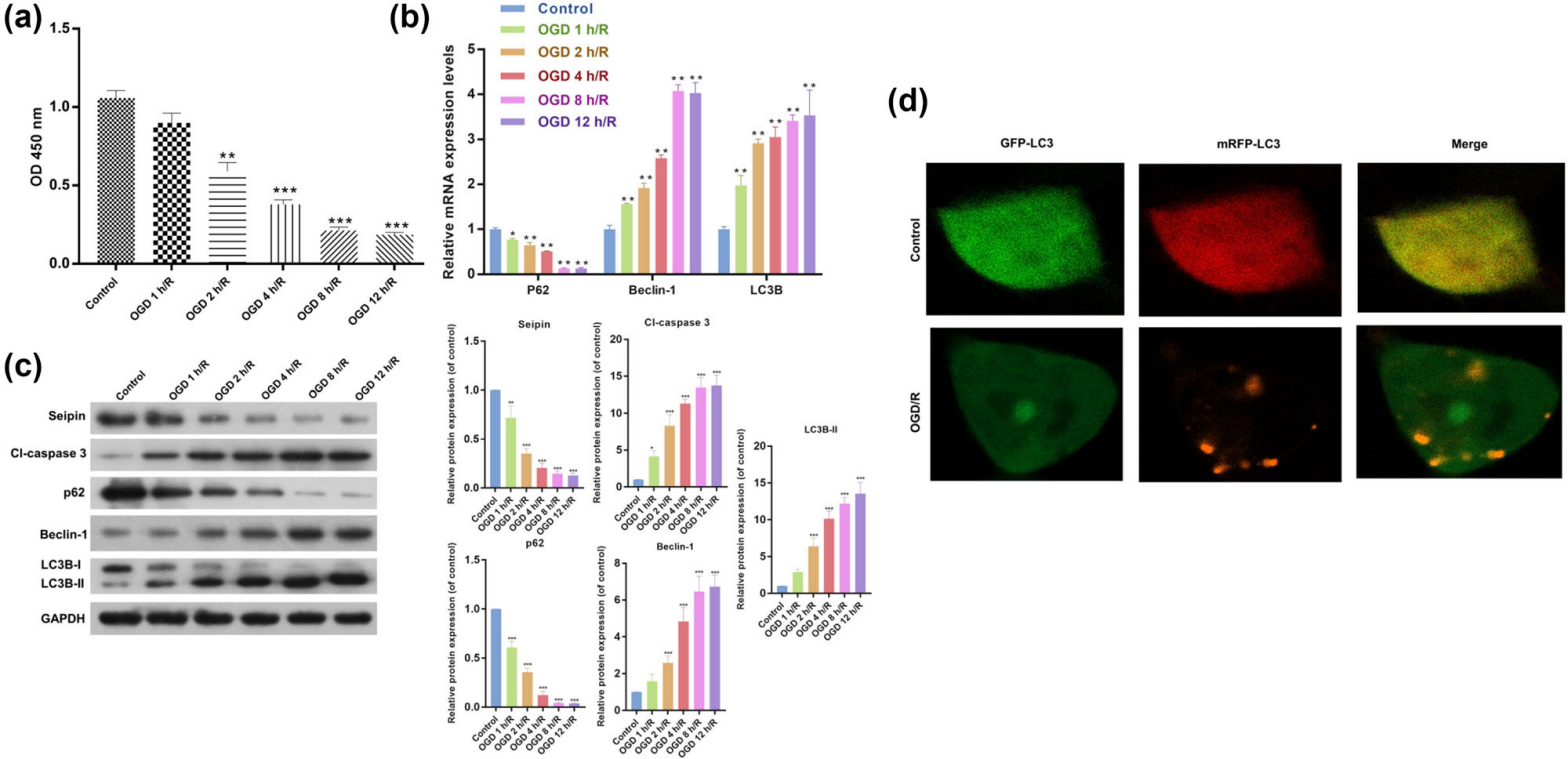
**Apoptosis and autophagy‐related proteins were notably altered in HT‐22 cells after OGD/R**. (a) CCK‐8 assay was utilized to identify the proliferation of HT‐22 cells, which were reoxygenated for 24 h after 1, 2, 4, 8, and 12 h of hypoxia. Results are presented as mean ± SD. **p < .01, ***p < .001 versus control. *n* = 3. (b) RT‐qPCR data displayed the changes of P62, Beclin‐1, and LC3B expressions. Results are presented as mean ± SD. *p < .05, **p < .01, ***p < .001 versus control. *n* = 3 (c) Western blot demonstrated the changes in Cl‐Caspase 3, P62, Beclin‐1 and LC3II/I expressions in HT‐22 cells, which were processed with 1, 2, 4, 8, and 12 h of hypoxia and 24 h of reoxygenation. (d) Representative images of HT22 cells transfected with Ad‐mCherry‐GFP‐LC3B adenovirus after treatment with OGD/R and analyzed by immunofluorescence. Green: FITC‐labeled LC3B; Red: Lyso‐Tracker‐labeled lysosomes. Results are presented as mean ± SD. *p < .05, **p < .01, ***p < .001 versus control. *n* = 3. The one‐way ANOVA was used for the comparison between multiple groups.

To investigate a feasible therapeutic target in cerebral I/R, we first established an OGD/R HT‐22 cell model. As show in Figure 1a, with an increase in hypoxia time, the viability of OGD/R‐induced HT‐22 cells gradually decreased. In summary, these data revealed that OGD/R treatment decreased the proliferative activity of HT‐22 cells.

### 3.2 Apoptosis and autophagy‐related proteins were notably altered in HT‐22 cells after OGD/R

Next, we examined changes in the expression of autophagy‐related genes in OGD/R HT‐22 cells. As displayed in Figure 1b, Beclin‐1 and LC3B expression was notably elevated, P62 expression was notably decreased in the OGD/R model group versus that in the control group, and the degree of change in these proteins gradually elevated as HT‐22 cells were deprived of oxygen for longer. We also found that OGD/R could result in remarkable upregulation of Cl‐Caspase 3, Beclin‐1, and LC3II/I expression and a noteworthy downregulation in P62 expression in HT‐22 cells (Figure 1c). To demonstrate the formation of autophagosomes, HT‐22 cells were transfected with Ad‐mCherry‐GFP‐LC3B adenovirus. As shown in Figure 1d, OGD induced significant autophagosome accumulation, which was characterized by GFP and RFP fluorescence signals (yellow). In summary, these findings confirmed that OGD/R treatment could significantly inhibit apoptosis and increase autophagy in HT‐22 cells. Based on the above results, we selected model cells deprived of oxygen for 4 h for the subsequent experiments.

### 3.3 Seipin overexpression induced cell viability and reduced apoptosis and autophagy‐related protein expression in OGD/R HT‐22 cells

**FIGURE 2:**
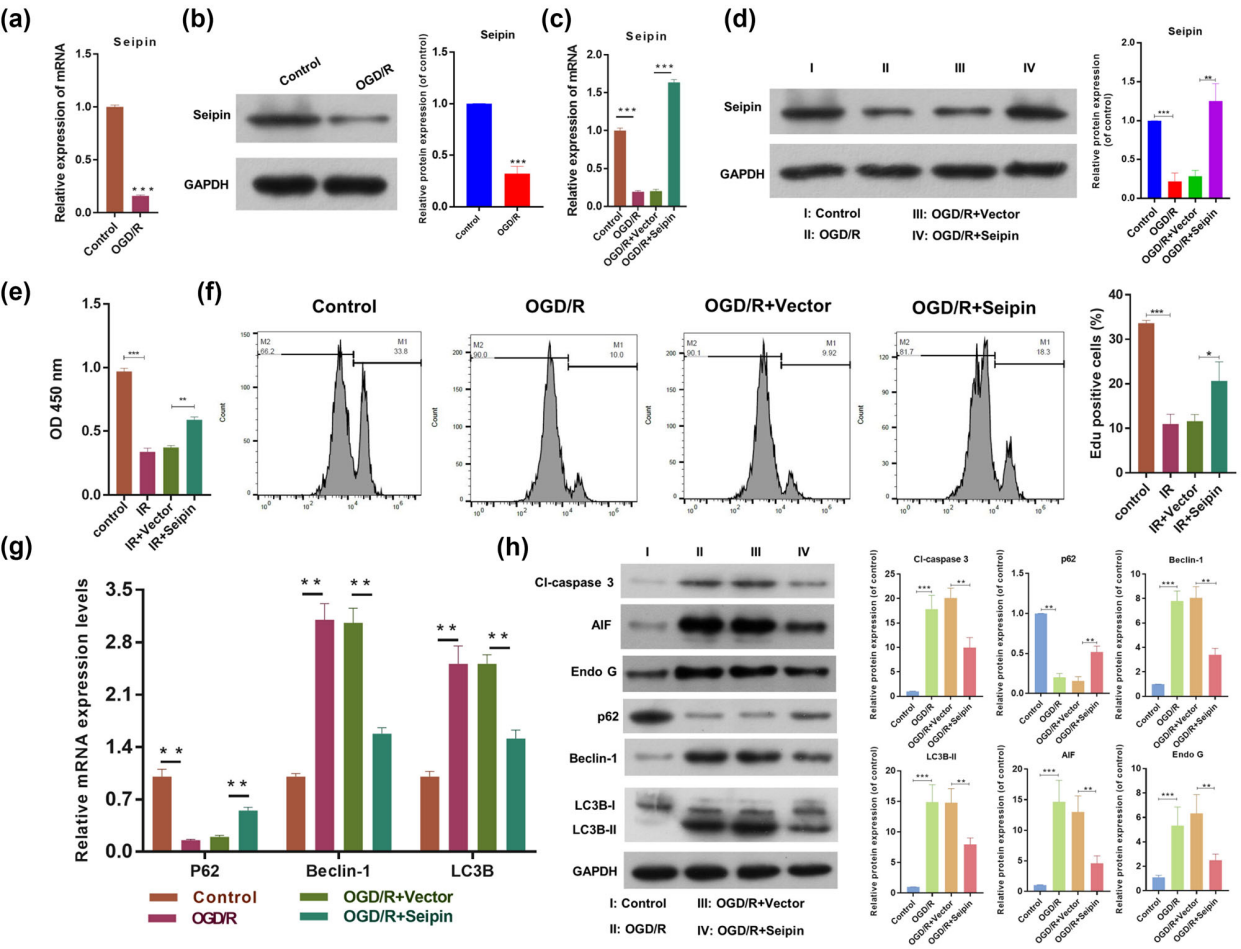
**Seipin overexpression evidently induced cell viability, reduced apoptosis and autophagy‐related proteins in HT‐22 cells after OGD/R**. (a) RT‐qPCR was applied to test Seipin expression in HT‐22 cells after OGD/R. Results are presented as mean ± SD. ***p < .001 versus control. *n* = 3 (b) The change of Seipin expression was assessed via western blot. Results are presented as mean ± SD. ***p < .001 versus control. *n* = 3. (c) RT‐qPCR and (d) Western blot were also adopted to test the expression change of Seipin, which were also overexpressed by Seipin. Results are presented as mean ± SD. ***p < .001 versus control. *n* = 3. (e) Cell proliferation was monitored with CCK‐8 after Seipin overexpression. Results are presented as mean ± SD. **p < .01, ***p < .001 versus control. *n* = 3. (f) Edu‐positive cells were confirmed through Flow Cytometer. Results are presented as mean ± SD. *p < .05, ***p < .001 versus control. *n* = 3. (g) RT‐qPCR analysis of P62, Beclin‐1, and LC3B expressions in the processed HT‐22 cells. Results are presented as mean ± SD. **p < .01 versus control. *n* = 3. (h) Western blot was also utilized to estimate the changes in Cl‐Caspase 3, P62, Beclin‐1, and LC3II/I expressions in the treated HT‐22 cells. Results are presented as mean ± SD. **p < .01, ***p < .001 versus control. *n* = 3. The Student's *t*‐test was used for the comparison between the two groups.

In addition, we discovered that seipin was downregulated in the OGD/R model group as compared to that in the control group (Figure 2a and b), indicating that seipin might affect certain biological functions of OGD/R HT‐22 cells. Thus, we overexpressed seipin in OGD/R‐treated HT‐22 cells. The results also presented that seipin overexpression markedly reversed the reduction in seipin expression (Figure 2c and d). Subsequently, CCK‐8 data presented that cell proliferation was notably weakened in the OGD/R HT‐22 cells as versus that in the control group, while seipin overexpression dramatically strengthened the cell proliferation in the OGD/R HT‐22 cells (Figure 2e). Flow cytometry data also presented that OGD/R distinctly reduced the EdU‐positive HT‐22 cells, while this reduction in the EdU‐positive cells could also be weakened by seipin (Figure 2f). Additionally, the results of RT‐qPCR signified that seipin overexpression notably lowered Beclin‐1 and LC3B expression and elevated P62 expression in HT‐22 cells under OGD/R (Figure 2g). The data also signified that seipin overexpression could observably downregulate Cl‐Caspase 3, Beclin‐1, and LC3II/I and upregulate P62 in OGD/R HT‐22 cells (Figure 2h). Thus, we suggest that seipin overexpression could accelerate viability and prevent apoptosis and autophagy in OGD/R HT‐22 cells.

### 3.4 Seipin overexpression prominently attenuated cerebral infarction degree, pathological brain injury, and apoptosis in MCAO rats

**FIGURE 3:**
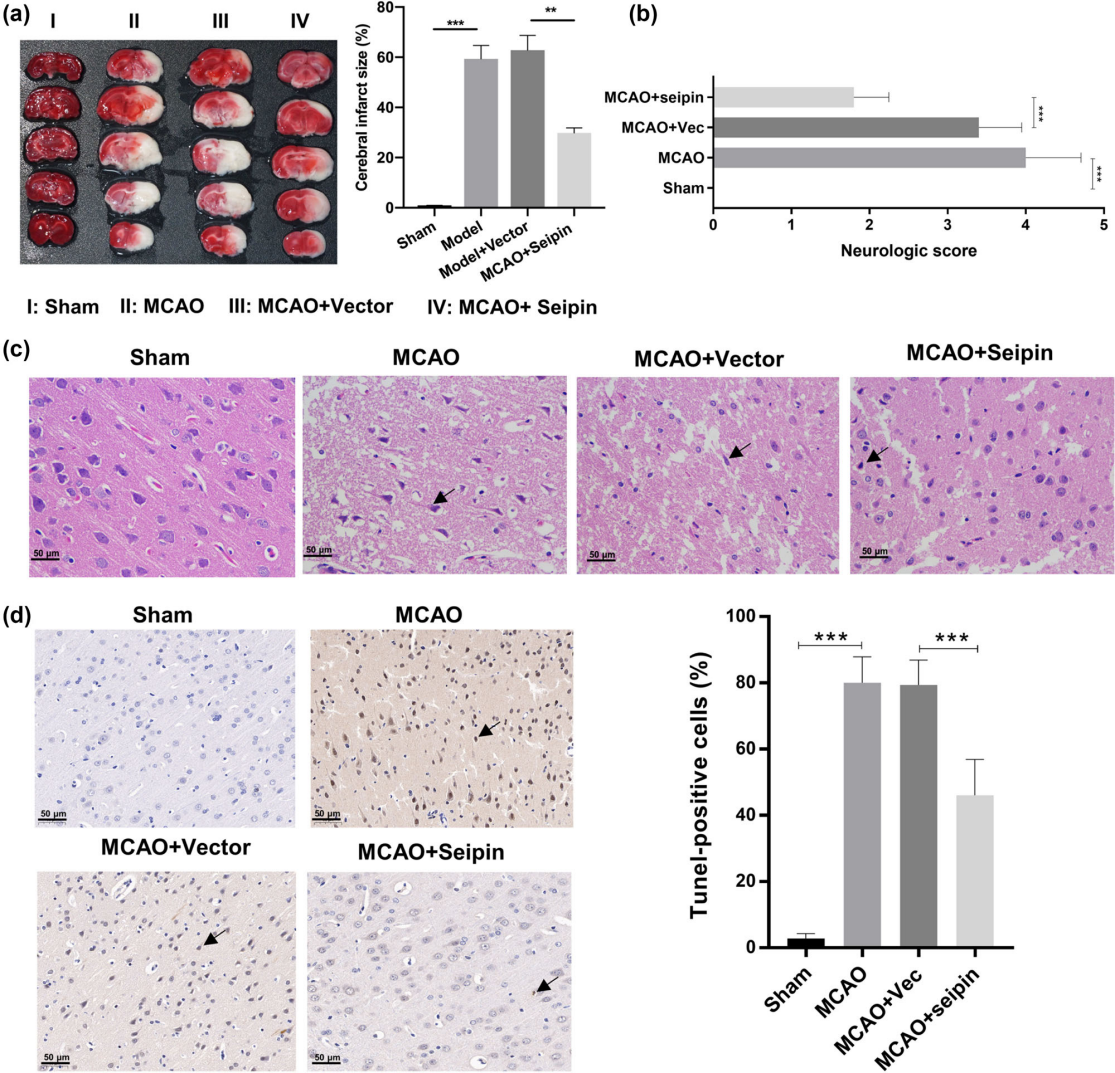
**Seipin overexpression prominently attenuated cerebral infarction degree, pathological brain injury, and apoptosis in MCAO rats**. MCAO rats were also established, and Seipin was overexpressed in the brain of model rats. (a) The degree of ischemic infarction was monitored through TTC staining in the brain of rats, and the infarct volume was also calculated. Results are presented as mean ± SD. **p < .01, ***p < .001 versus sham group. *n* = 10. (b) The neurologic score was assessed by Siegal method. Results are presented as mean ± SD. ***p < .001 versus sham group. *n* = 10. (c) The pathological injury of brain tissues was confirmed using H&E staining. The necrotic cells indicated by black tips. Magnification, 200×, scale bar = 50 μm. *n* = 10. (d) TUNEL assay was adopted to evaluate the apoptosis in each group. Magnification, 200×, scale bar = 50 μm. *n* = 10. The Student's *t*‐test was used for the comparison between the two groups.

We investigated the possible impact of seipin in vivo. First, we established MCAO rats, which were then overexpressing seipin. The TTC staining data signified that versus that of sham‐operated rats, the infarct volume (white area) was notably elevated in the MCAO rats, while this increase in infarct volume could also be attenuated by seipin overexpression in the MCAO rats (Figure 3a). And in line with the Siegal method, the neurologic score was noticeably elevated in MCAO group versus that in sham group, and the elevation of neurologic score also could be reversed by Seipin overexpression in MCAO rats (Figure 3b). In addition, histopathological assessment of brain coronal sections with H&E staining results represented that in the sham group, the structure of the brain tissue was normal, the cells were arranged neatly, the outline of cells was clear, and the neurons were not necrotic; in the model group, the structure of the brain tissue was unclear and disordered, and severe necrosis was observed; in the Seipin overexpression group, the morphology of neurons was dramatically improved (Figure 3c). Besides, TUNEL results denoted that the brain apoptosis was dramatically enhanced in MCAO group versus that in sham group, and the enhancement of brain apoptosis also could be notably attenuated by Seipin overexpression in MCAO rats (Figure 3d). Overall, our results revealed that seipin overexpression ameliorated brain injury in the MCAO rats.

### 3.5 Overexpression of seipin markedly prevented apoptosis and autophagy through the examination of related proteins in MCAO rats

**FIGURE 4:**
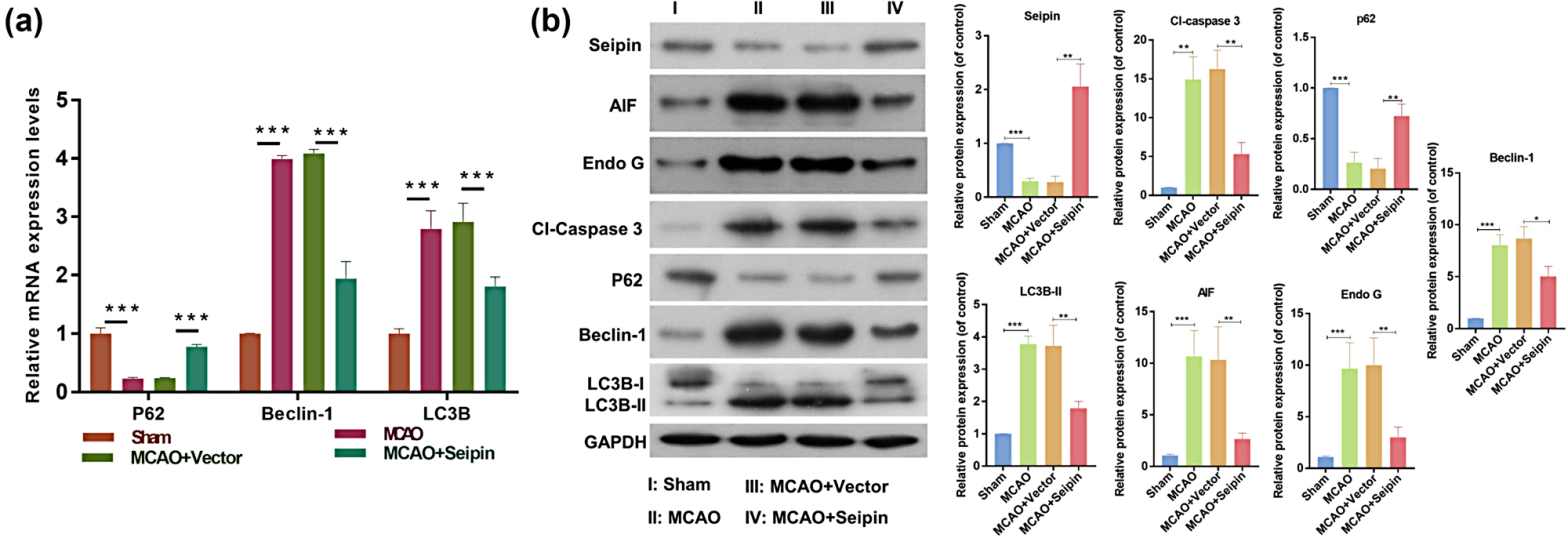
**Overexpression of Seipin markedly prevented apoptosis and autophagy through the examination of related proteins in MCAO rats**. (a) RT‐qPCR was utilized to determine the impact of Seipin overexpression on p62, Beclin‐1, and LC3B in MCAO rats, which was overexpressed by Seipin. Results are presented as mean ± SD. ***p < .001 versus sham group. *n* = 3. (b) Western blotting analysis of Seipin, Cl‐Caspase 3, P62, Beclin‐1 and LC3II/I expressions in the brain tissues, and relative protein levels were quantified based on gray values. Results are presented as mean ± SD. *p < .05, **p < .01***p < .001 versus sham group. *n* = 3. The Student's *t*‐test was used for the comparison between the two groups.

Similarly, we investigated the influence of seipin on apoptosis‐ and autophagy‐related proteins in MCAO rats. In mRNA level, Beclin‐1 and LC3B expression was prominently elevated and P62 expression was notably diminished in the MCAO model group, while the changes in these three genes could also be memorably attenuated by seipin overexpression in MCAO rats (Figure 4a). Simultaneously, we proved that overexpression of seipin also notably decreased Cl‐Caspase 3, Beclin‐1 and LC3II/I expression and elevated P62 expression in the MCAO rats at protein level (Figure 4b). Overall, we verified that seipin overexpression could also reduce apoptosis and autophagy in the brains of MCAO model rat.

## 4 DISCUSSION

Ischemic cardiovascular disease is a major cause of death and disability globally (Lanzino & Brown, 2014) and CI/RI is an inevitable pathophysiological process in the treatment of ischemic CVD (Wang et al., 2020b). Cerebral edema secondary to CI/RI is the most serious complication and the primary cause of death in patients with ischemic stroke (Yuan et al., 2021). Currently, although some achievements have been made in CI/RI therapy, most are symptomatic treatment (Wu et al., 2018). While the mechanism underlying CI/RI formation was not yet been fully elucidated. The OGD/R is a classical model used to explore the CI/RI in vitro (Sun et al., 2020). To verify the possible therapeutic targets of CI/RI, we constructed an OGD/R HT‐22 cell model (Che et al., 2022). Our results indicated that cell viability was notably reduced in OGD/R HT‐22 cells. The MCAO model is also a classical model in CI/RI research, which can simulate the ischemic process of the human brain (Wu et al., 2017). We constructed a CI/RI model based on the MCAO method. Our results presented that in MCAO rats, the cerebral infarction area was notably elevated, and there was obvious necrosis of brain tissue, including elevated cell space, edema, and degree of cell proliferation. Thus, we successfully established in vitro and in vivo CI/RI models.

Tissue injury caused by ischemia‐reperfusion is divided into ischemia and reperfusion injury (İşgüder et al., 2021; Saidi & Kenari, 2014). During reperfusion of ischemic tissue, local inflammation and the production of ROS dramatically increase, leading to secondary injury (İşgüder et al., 2021; Wu et al., 2018). Then, I/RI causes apoptosis, autophagy, and necrosis (Wu et al., 2018). Apoptosis is a crucial mechanism associated with CI/RI. During cerebral ischemia, the death of neurons around the ischemic central area is mainly due to apoptosis (Gong et al., 2017). Members of the caspase family play a leading role in both mitochondrial‐dependent and nondependent apoptosis, and Caspase‐3 is one of the vital indicators adopted to test apoptosis (Fan et al., 2005). In our study, we also discovered that Cl‐Caspase 3 was upregulated in the MCAO rats and OGD/R HT‐22 cells. Therefore, we speculated that apoptosis is enhanced during CI/RI.

Autophagy is a molecular biological process in cells (Mizushima & Komatsu, 2011; Wang et al., 2022). Autophagy can be induced by external factors (e.g., stress injury, lack of growth factors, amino acid deficiency) or internal factors (e.g., endoplasmic reticulum stress (ERS), mitochondrial damage, abnormal lipid metabolism) (Parzych & Klionsky, 2014). Through autophagy, cells can recycle intracellular amino acids and other energy products under harmful conditions such as hunger and stress to achieve cell survival (Glick et al., 2010). Currently, autophagy detection methods primarily involve direct (observation of autophagy morphology) and indirect methods (determination of autophagy characteristic proteins). Beclin‐1 activation can initiate autophagy and induce the formation of autophagic lysosomes, which can serve as a marker of autophagy initiation (Tran et al., 2021). The LC3 is an autophagy‐related protein homologous to Atg8 in yeast, and the LC3‐II is a marker of autophagosome formation (Tanida et al., 2008). During autophagosome formation, cytoplasmic LC3 (LC3‐I) is transformed into autophagosome membrane type (LC3‐II) (Runwal et al., 2019). P62 is also a well‐known autophagy marker. During autophagy, autophagosomes and lysosomes fuse to degrade misfolded proteins, and P62 expression decreased (Lamark et al., 2017). Autophagy, a pathway to clear the damaged mitochondria, plays a key role in CI/RI (Gong et al., 2018). Mitochondrial dysfunctions in the CI/R are vital causes of nerve cell death (Cai et al., 2021). Recent studies have displayed that autophagy plays a protective role in ischemia preconditioning, but it may have a different effect when I/RI occurs (Galkin, 2019; Yang et al., 2019). Whether the activation of autophagy is a protective mechanism or a mechanism of injury is still unclear. The autophagy‐induced effects facilitate the necrotic and apoptotic cascades, and thereby results in a cell death (Wang et al., 2018b). Autophagy is called type II programmed cell death or apoptosis. Autophagy seems to be a double‐edged sword. Whether autophagy is beneficial or deleterious depends on the rate of autophagy induction and the duration of autophagy activation (Zhang et al., 2020b). It was discovered that mitochondrial autophagy could be repressed 14 days after I/R (Wang et al., 2020a). In our study, we confirmed that Beclin‐1 and LC3II/I could be upregulated and P62 could be downregulated in OGD/R HT‐22 cells and OGD/R (ischemia for 4 h and reperfusion for 24 h) MCAO rats, indicating that autophagy was notably enhanced during CI/RI.

Seipin is located in the ER, and mutated seipin can activate the cellular unfolded protein response and induce ERS‐mediated apoptosis (Ren et al., 2021). Moreover, seipin can activate ERS and cause muscle spasm and atrophy (Ramos‐Lopes et al., 2021). Mutated seipin can also elevate ERS proteins (GRP78 and CHOP) and accelerate apoptosis (Wu et al., 2021). N88S/S90L mutations in seipin and seipin knockout rats also displayed mild ERS in rats (Fei et al., 2011). In our study, we discovered that seipin overexpression promoted viability and repressed apoptosis and autophagy in OGD/R HT‐22 cells and MCAO rats.

The levels of apoptosis and autophagy‐related proteins such as Caspase 3, P62, Beclin‐1, LC3II/I, etc. were confirmed by western blot in MCAO model or OGD/R cell model. But the mechanisms of the effect of seipin on apoptosis and autophagy had not been observed. And the mechanisms of the effect of seipin on apoptosis and autophagy needs for further research. We will search for it in the further research.

## 5 CONCLUSIONS

Our results showed that seipin overexpression relieved CI/RI and suppressed apoptosis and autophagy‐related proteins in OGD/R HT‐22 cells and MCAO rats. Therefore, seipin may be a potential genetic target for the future treatment of CI/RI.

## AUTHOR CONTRIBUTIONS

Xiaoxi Zhu, Xiaoqiong An, Ming Chen, and Wenfeng Yu conducted literature research, analyzed data, experimented, and prepared the manuscript. Dongfeng Guo, Peng Xie, Bi Wang, and Zhi Huang designed the project, experimented, and edited the manuscript. Xiaoxi Zhu collected data, wrote the manuscript, and analyzed the data.

## CONFLICT OF INTEREST STATEMENT

The authors declare that they have no competing interests.

### ETHICS STATEMENT

This experiment was approved by Experimental Animal Care and Usage Committee of Guizhou Medical University. The research was conducted in compliance with the Declaration of Helsinki. All animal procedures were conducted with the NIH guidelines. All methods were carried out in accordance with relevant guidelines and regulations. This study was carried out in compliance with the ARRIVE guidelines.

### PEER REVIEW

The peer review history for this article is available at https://publons.com/publon/10.1002/brb3.3195.

## Data Availability

The data used to support the findings of this study are available from the corresponding author upon request.
